# Chicken DNA Sensing cGAS-STING Signal Pathway Mediates Broad Spectrum Antiviral Functions

**DOI:** 10.3390/vaccines8030369

**Published:** 2020-07-09

**Authors:** Shuangjie Li, Jie Yang, Yuanyuan Zhu, Xingyu Ji, Kun Wang, Sen Jiang, Jia Luo, Hui Wang, Wanglong Zheng, Nanhua Chen, Jianqiang Ye, François Meurens, Jianzhong Zhu

**Affiliations:** 1 Comparative Medicine Research Institute, Yangzhou University, 88 South University Avenue, Yangzhou 225009, China; d160106@yzu.edu.cn (S.L.); mx120170736@yzu.edu.cn (J.Y.); Fanstisticbaby@163.com (Y.Z.); mx120170691@yzu.edu.cn (X.J.); mx120170734@yzu.edu.cn (K.W.); jiangsen8888@163.com (S.J.); luojia8888888@163.com (J.L.); 15104575603@163.com (H.W.); wanglongzheng@yzu.edu.cn (W.Z.); hnchen@yzu.edu.cn (N.C.); jqye@yzu.edu.cn (J.Y.); 2 College Veterinary Medicine, Yangzhou University, 88 South University Avenue, Yangzhou 225009, China; 3 Jiangsu Co-Innovation Center for Prevention and Control of Important Animal Infectious Diseases and Zoonoses, Yangzhou University, 88 South University Avenue, Yangzhou 225009, China; 4 Joint International Research Laboratory of Agriculture and Agri-Product Safety, Yangzhou 225009, China; 5 BIOEPAR, INRAE, Ecole Nationale Vétérinaire Oniris, CEDEX 3, 44307 Nantes, France; francois.meurens@oniris-nantes.fr

**Keywords:** cGAS-STING, DNA sensors, signaling pathway, antiviral, chicken

## Abstract

The innate DNA sensing receptors are one family of pattern recognition receptors and play important roles in antiviral infections, especially DNA viral infections. Among the multiple DNA sensors, cGAS has been studied intensively and is most defined in mammals. However, DNA sensors in chickens have not been much studied, and the chicken cGAS is still not fully understood. In this study, we investigated the chicken cGAS-STING signal axis, revealed its synergistic activity, species-specificity, and the signal essential sites in cGAS. Importantly, both cGAS and STING exhibited antiviral effects against DNA viruses, retroviruses, and RNA viruses, suggesting the broad range antiviral functions and the critical roles in chicken innate immunity.

## 1. Introduction

The innate immune system, as the first line of host defense, expresses various pattern recognition receptors (PRRs) to recognize multiple danger signals from pathogens or cellular damage, which are the pathogen associated molecular patterns (PAMPs) and damage associated molecular patterns (DAMPs), respectively [1,2]. The innate immune pattern recognition receptors (PRRs) comprise different families, including membrane bound Toll-like receptors (TLRs) and C-type lectin-like receptors (CLRs), cytosolic RIG-I-like receptors (RLRs), NOD-like receptors (NLRs), and cytosolic DNA receptors (CDRs). Upon engagement by PAMPs or DAMPs, the innate immune PRRs trigger intracellular cellular signaling pathways to activate either gene transcription or protease-dependent cytokine maturation. The cellular events lead to the production of antiviral interferons (IFNs), proinflammatory cytokines, and chemokines to fight pathogens or achieve homeostasis, which also influences subsequent adaptive immunity [2].

Among the five PRR families, TLRs, RLRs, and CDRs play a major role in recognizing the virus infections through sensing the viral nucleic acids, DNA and RNA, which are the most abundant signatures in viral infected cells [3,4]. Specifically, TLR members TLR3, TLR7/8 and RLRs members RIG-I, MDA5 are the sensors for viral RNAs including single stranded RNA (ssRNA by TLR7/8) and double stranded RNA (dsRNA by TLR3, RIG-I, and MDA5). On the other hand, the TLR9 and broad range of CDR family members are the sensors of viral DNAs, which have attracted more attentions in recent years for antiviral immunity.

DNA sensor TLR9, an endosomal/ER membrane receptor and the first discovered DNA sensor, recognizes endolysosomal under-methylated CpG DNA to activate transcription factors IRF7 and NF-ĸB and stimulate type I IFN production [5]. Two CDR members, DExD/H-box helicases DHX36 and DHX9, are required in plasmacytoid dendritic cells (pDCs) as accessory receptors for TLR9 dependent IFNα and TNF-α productions, respectively [6,7]. Additionally, other CDR family members include DAI, AIM2, IFI16, RNA Pol III, LRRFIP1, DDX41, DNA-PK, MRE11, cGAS, and STING [7].

DAI (or ZBP-1), the first discovered CDR, was a identified as an IFN inducer through IRF3 and NF-κB activations, but later on, its role as a DNA sensor became controversial [8]. AIM2 and IFI16 both belong to PYHIN family proteins containing Pyrin and DNA binding HIN domains. AIM2 recruits downstream adaptor ASC to mediate inflammasome formation, leading to activation of caspase-1 and proteolytic cleavage and maturation of the proinflammatory cytokines IL1β and IL18 [7,9]. IFI16 was the first reported STING-dependent DNA sensor mediating IFN induction [10]. Moreover, the nuclear IFI16 also engages in inflammasome formation [11], but the mechanisms by which IFI16 initiates signal to both STING and inflammasome are still unknown [7]. RNA polymerase III (Pol III) was described as a DNA sensor because of its transcription of AT-rich dsDNA into 5-triphosphate RNA, which in turn activates RIG-I leading to IFNβ induction [12,13]. LRRFIP1 was reported to bind both double stranded (ds) DNA and dsRNA and then activate β-catenin, a co-activator of master transcription factor IRF3 to increase IFNβexpression [14]. DDX41, another DExD/H-box helicase, was shown to bind with DNAs including viral DNA and bacterial cyclic dinucleotides (CDNs) to activate STING/TBK1 dependent IRF3 and NF-κB, and subsequent cytokine production [15]. DNA-PK and MRE11 are both nuclear DNA damage sensors. Both protein complexes in cytosol were demonstrated to be involved in DNA sensing and trigger STING dependent cytokine production [16,17].

STING (also called MITA, MPYS, and ERIS), which was discovered in 2008 by several groups independently [18,19,20,21], acts as the signaling adaptor for DNA sensing pathways. STING also directly recognizes CDNs, such as bacterial c-di-GMP and mammalian second messenger cGAMP, to induce a type I IFN response [22,23]. cGAS was identified in 2013 as a cytosolic DNA sensor, which, upon dsDNA activation, utilized substrates ATP and GTP to synthesize second message 2′3′-cGAMP, directly activating STING signaling [24]. Since its discovery, the defined cGAS-cGAMP-STING pathway has been extensively investigated, especially in antiviral research [25].

Chickens have distinct species specificity in the DNA sensing pathways from mammals. For example, chickens and other avian species, such as duck and goose, do not have TLR9, but instead have a functional ortholog named TLR21 with very little amino acid sequence similarity to mammalian TLR9 [26]. Second, DAI, DHX9, and PYHIN family members AIM2, IFI16 are all absent in chickens and other avian species [27]. On the other hand, DDX41 and cGAS-STING pathways exist in chickens and other avian species. The DDX41 sequences are highly conserved between avian and mammals [27], and chicken and duck DDX41 have been characterized both showing STING dependent signaling as mammalian DDX41 [28,29]. Despite the low sequence identity to mammalian counterparts, the avian cGAS-STING pathway functions similarly and plays a critical role in restricting DNA virus infections [27,30]. Several studies from one group showed that Marek’s disease virus (MDV) has evolved different immune evasion mechanisms against chicken cGAS-STING pathway [31,32,33], whereas another study suggested that chicken cGAS is responsible for recognition of fowl adenovirus [34]. However, the chicken cGAS-STING pathway has not been fully understood. Here, we investigated the chicken cGAS-STING signal axis, its synergistic signal activity, species-specificity, and the signal essential sites. Particularly, we found that both chicken cGAS and STING have broad spectrum antiviral functions.

## 2. Materials and Methods

### 2.1. Cells, Reagents and Viruses

HEK-293T and DF-1 cells were cultured in Dulbecco’s modified Eagle medium (DMEM, Hyclone Laboratories, Logan, UT, USA) containing 10% fetal bovine serum (FBS) and 100 IU/mL of penicillin plus 100μg/mL streptomycin. Chicken macrophage HD11 cells were maintained in RPMI 1640 (Hyclone Laboratories) supplemented with 10% FBS. TransIT-LT1 Transfection Reagent for HD11 and DF-1 cells was purchased from Mirus Bio (Madison, WI, USA). PrimerSTAR Max DNA Polymerase was bought from TAKARA (Beijing China). Restriction endonucleases and T4 DNA ligase (M0203S) were all purchased from New England Biolabs (Beijing, China). Gateway^TM^ LR Clonase^TM^ II Enzyme mix and lipofectamine^TM^ 2000 were from ThermoFisher Scientific (Shanghai, China). TRIpure Reagent for RNA extraction was from Aidlab (Beijing, China). EasyScript Reverse Transcriptase, 2×EasyTag PCR SuperMix, BluePlus Protein Marker, anti-HA mouse mAb, anti-FLAG mouse mAb, Horseradish peroxidase (HRP) anti-mouse IgG, HRP anti-rabbit IgG, and TransDetect Double-Luciferase Reporter Assay Kit were all from Transgen Biotech (Beijing, China). Forty-five bp (45bp) dsDNA (TACAGATCTACTAGTGATCTATGACTGATCTGTACATGATCTACA) as a DNA agonist was synthesized by GENEWIZ (Shouzhou, China). Agonist pcDNA3.1 was prepared by EndoFree Mini Plasmid Kit (TianGen, China). Vaccinia Viruses (Western-Reserve Vvt7 strain, VACV and Western-Reserve VV strain, SMV) were gifts from Prof. Zhengfan Jiang of Peking University China. Herpes Simplex Virus-1 (HSV-1, KOS strain, in which VP26 was fused with GFP) and Vesicular Stomatitis Virus (VSV-GFP) were both gifts from Dr. Tony Wang of SRI International USA. Sendai virus (SeV-GFP) was a gift from Dr. Feng Ma of Suzhou Institute of Systems Medicine, China. Encephalomyocarditis Virus (EMCV, tissue culture adapted) was purchased from ATCC (VR-129B). Recombinant chicken Newcastle Disease Virus (NDV-RFP) was a gift from Prof. Xiufan Liu of Yangzhou University, and Avian Leukemia Virus (ALV-A) was provided by Dr. Jianqiang Ye of Yangzhou University.

### 2.2. Molecular Cloning and Gene Mutations

Total RNA was extracted from DH11 cells using TRIpure reagent, and chicken cGAS (XM_419881) and STING (NM_001305152) open reading frames (ORFs) were amplified by RT-PCR using the newly designed primers shown in Table S1. The chcGAS PCR product was digested with *Nco*I/*Sma*I and cloned into the *Nco*I/*EcoR*V sites of Gateway entry vector pENTR4-3FLAG and pENTR4-2HA, which was adapted from pENTR4 (Addgene) by inserting 3FLAG and 2HA sequences behind *EcoR*V to express C-terminal FLAG and HA, respectively. The chSTING PCR product was digested with *Sal*I/*EcoR*V and cloned into the same sites of pENTR4-3FLAG. The sequence confirmed FLAG, HA-tagged chcGAS, and FLAG tagged chSTING were transferred from pENTR4 vectors to Destination vectors pDEST47 (Addgene, Watertown, MA, USA) by LR recombination to obtain the final pcDNA recombinant expression vectors.

The conserved amino acids across chcGAS, human cGAS, mouse cGAS, and porcine cGAS were identified, and the potential signaling essential sites were mutated. The chcGAS mutation PCR primers were designed using QuickChange Primer Design method (https://www.agilent.com) (Table S2). The mutation PCR was performed using PrimerSTAR Max DNA Polymerase and pENTR4-chcGAS-3FLAG as the template. The PCR products were digested with *Dpn I* and transformed into competent DMT *E.coli* to proliferate the mutant plasmids. The sequence confirmed chcGAS mutants were all transferred from pENTR4 plasmids into the Destination vectors pDEST47 by LR recombination to obtain the mutant chcGAS expression vectors.

### 2.3. CRISPR gRNA Design, gRNA, and Gene Expressing Lentiviruses and Stable HD11 Cells

The CRISPR gRNAs targeting chcGAS and chSTING were designed using the web tool from Benchling (www.benchling.com). Four gRNAs were chosen based on the predicted high scores for chcGAS and chSTING, respectively, which are shown in Table S3. The annealed gRNA DNA pairs were ligated with *BsmB1* digested lentiCRISPRv2 vector (Addgene), and the efficacies of these gRNA expressing lentiviral vectors targeting chcGAS and chSTING were examined to choose the best gRNAs. The gRNA expressing lentiviruses were generated by co-transfecting lentiCRISPRv2-gRNAs with package plasmids psPAX2 and pMD2.G into 293T cells using Lipofectamine 2000. The supernatants containing the chcGAS and chSTING gRNA expressing lentiviruses were used to infect the HD11 cells, respectively. Next, the infected HD11 cells were selected with 0.5–1.0 μg/mL puromycin, and the survived cells were chGAS KO, chSTING KO, and CRISPR vector control stable HD11 cells, respectively. The pENTER4-chcGAS-3FLAG, pENTER4-chSTING-3FLAG, and control vector pENTR4-3FLAG were subjected to LR reactions with pDEST lentiCMV puro (Addgene), respectively, to obtain the lentiviral expression vectors. The gene expressing lentiviruses were similarly packaged in 293T cells and used to infect HD11 cells. The infected cells were selected with puromycin, and the survived cells were chGAS OE, chSITNG OE, and control pCMV stable HD11 cells, respectively.

### 2.4. Co-Immunoprecipitation and Western Blot Analysis

For co-immunoprecipitation, the cleared cell lysates from transfected cells were incubated with 1 μg anti-FLAG antibody at 4 °C overnight, with shaking. Then, they were further incubated with Protein A/G PLUS-Agarose (sc-2003, Santa Cruz Biotechnology, Dallas, TX, USA) for 23 h. The agarose was washed three times followed by elution with 40 μL 2 × SDS sample buffer. The elutions and inputs from clear cell lysates were both subjected to Western-blot analysis. Cell lysates and precipitated samples were resolved on 10% SDS-polyacrylamide gels in the presence of 2-mercaptoethanol. The protein bands on gels were transferred onto PVDF membranes and the membranes were blocked with 5% non-fat dry milk Tris-buffered saline pH 7.4 with 0.1% Tween-20 (TBST), incubated with anti-FLAG and anti-HA mouse mAbs. After washing with TBST, the membranes were incubated with HRP-conjugated goat anti-mouse IgG (1:10,000 dilutions). The bound secondary antibody signals were detected with enhanced chemiluminescence (ECL) substrate (Tanon, China) and visualized by Western blot imaging system (Tanon Science & Technology Co.,Ltd., Shanghai, China).

### 2.5. Con-Focal Fluorescence Microscopy

HEK-293T cells grown in 24-well culture plates (2 × 10^5^ cells/well) were transfected with chcGAS-2HA and chSTING-3FLAG using Lipofectamine 2000. Twenty-four hours later, the transfected cells were fixed with 4% paraformaldehyde for 20 min, permeabilized with 0.5% Tritonx-100 for 20 min, and blocked in 5% BSA for 30 min at RT, sequentially. The cells were next incubated with anti-HA rabbit pAb (Cell Signaling Technology, Danvers, MA, USA) and anti-FLAG mouse mAb (1:500 each) overnight at 4 °C, then the second DyLight^TM^ 488 Conjugated Goat Anti-rabbit IgG and DyLight^TM^ 594 Conjugated Goat Anti-mouse IgG (Invitrogen, Carlsbad, CA, USA, 1:800 each) at RT for 1 h. After washing with PBS, the cells were counter-stained with 0.5μg/mL DAPI for 15 min and the coverslips sealed with nail polish. Lastly, the cells were visualized under laser-scanning confocal microscope (LSCM, Leica SP8, Solms, Germany) at the excitation wavelengths 340 nm, 488 nm, and 594 nm, respectively.

### 2.6. Promoter Driven Luciferase Reporter Gene Assays

293T cells grown in 96-well plates (3 × 10^4^ cells/well) were co-transfected using Lipofectamine 2000 with ISRE-luciferase reporter or ELAM (NF-κB)-firefly luciferase (Fluc) reporter (10 ng/well) and β-actin *Renilla* luciferase (Rluc) reporter (0.2 ng/well), together with the indicated plasmids or vector control (5–40 ng/well). HD11 cells were similarly transfected with chicken IFNβ Fluc [35] using TransIT-LT1 Transfection Reagent. The total DNA per well was normalized to 50 ng by adding empty vector. About 36 h post-transfection, the cells were harvested, lysed, and both Fluc and Rluc activities were sequentially measured using the TransDetect Double-Luciferase Reporter Assay Kit. The results were expressed as fold induction of ISRE or ELAM (NF-κB)-Fluc compared with that of vector control after Fluc normalization by corresponding Rluc.

### 2.7. RT-Quantitative PCR

293T and HD11 cells grown in 24-well plates (3 × 10^5^ cells) were subjected to different treatments. Fluorescent virus infected HD11 stable cells were monitored by fluorescence microscopy before cell harvest and the florescence images were taken to show the virus replications. The treated cells were collected and RNA extracted with TRIpure reagent. The extracted RNA was reverse transcribed into cDNA with EasyScript Reverse Transcriptase, and then the target gene expressions were measured by quantitative PCR (qPCR) with 2× EasyTag PCR SuperMix using StepOnePlus equipment (Applied Biosystems). The qPCR program is denaturation at 94 °C for 30 s, followed by 40 cycles of 94 °C for 5 s and 60 °C for 30 s. The qPCR primers for hIFN-β, hISG56, hIL-1β, hIL8, hRPL32, chIFNβ, chOASL, chIL-1β, chIL-8, and chGAPDH are shown in Table S4, where the hRPL32 and chGAPDH are human and chicken housekeeping genes, respectively. The viral gene qPCR primers for VACV/SMV (C11R genes), VSV (Glycoprotein gene), SeV (GFP gene), EMCV (Polyprotein gene), NDV (F gene), and ALV-A (Env gene) were also given in Table S4. The transcriptional levels of cellular downstream genes and viral genes were calculated using ΔΔC_T_ method.

### 2.8. Statistical Analysis

The representative experimental data from three similar experiments were shown in graphs as the mean ± SD of duplicate wells. The statistical analysis was performed using the software GraphPad Prism 5.0 and the Student’s *t*-test was carried out when relevant. The signs “*”, “**”, “***” in the graphs denote *p* < 0.05, *p* < 0.01 and *p* < 0.001, respectively.

## 3. Results

### 3.1. Characterization of Chicken (ch) cGAS and STING Signaling Activities

**Figure 1:**
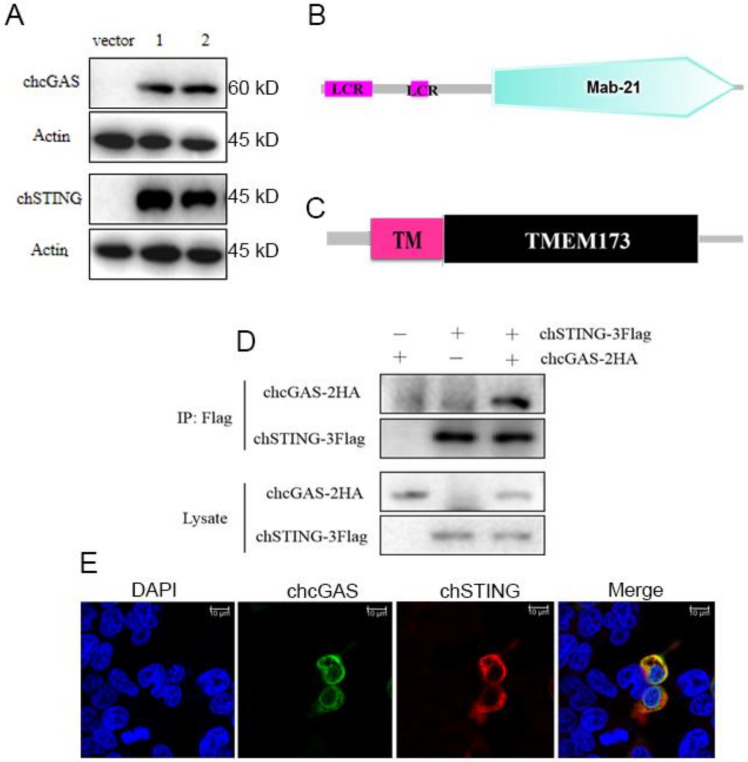
The structures, expressions, and interaction of chcGAS and chSTING. (**A**) The pcDNA-chcGAS-3FLAG (clones 1 and 2) and pcDNA-chSTING-3FLAG (clones 1 and 2) plus pcDNA3.1 (vector) were transfected into 293T cells in 24-well plate (3 × 10^5^ cells/well) for 48 h. The cells samples were detected by Western-blotting with anti-FLAG mAb. (**B,C**) The predicted chcGAS and chSTING protein structure and domains predicted by SMART software. LCR: low complexity region, TM: transmembrane region (28aa–50aa). (**D**) chcGAS-HA and chSTING-FLAG were co-transfected into 293T cells in 6-well plate (7 × 10^5^ cells/well), and the cell lystes were immunoprecipitated with anti-FLAG mAb and subjected to Western-blotting using the indicated antibodies. (**E**) chcGAS-HA and chSTING-FLAG were co-transfected into 293T cells in 24-well plate, and the cells were examined for co-localization by con-focal fluorescence microscopy.

Overall, chcGAS and chSTING both have low amino acid sequence identities (<50%) to mammalian human, mouse, and porcine counterparts (Table 1). chcGAS and chSTING coding genes were cloned, and their expressions in transfected 293T cells were examined by Western-blotting. The FLAG tagged chcGAS and chSTING were expressed as proteins of 60kDa and 45kDa, respectively, as expected (Figure 1A). The chcGAS comprises 438 amino acids and contains the conserved Mab-21 domain (AAs 129–429) predicted by online SMART software (http://smart.embl-heidelberg.de) (Figure 1B), whereas chSTING has 379 amino acids and contains the predicted conservative TMEM173 domain (AAs 50–342) (Figure 1C). Co-immunoprecipitation assay showed that there was interaction between chcGAS-HA and chSTING-FLAG proteins in transfected 293T cells (Figure 1D). Cellular co-localization also supported the interaction between chcGAS and chSTING (Figure 1E). Previously, we also found there was interaction between porcine cGAS and STING proteins, despite no requirement of this interaction for STING function and signaling.

**Figure 2:**
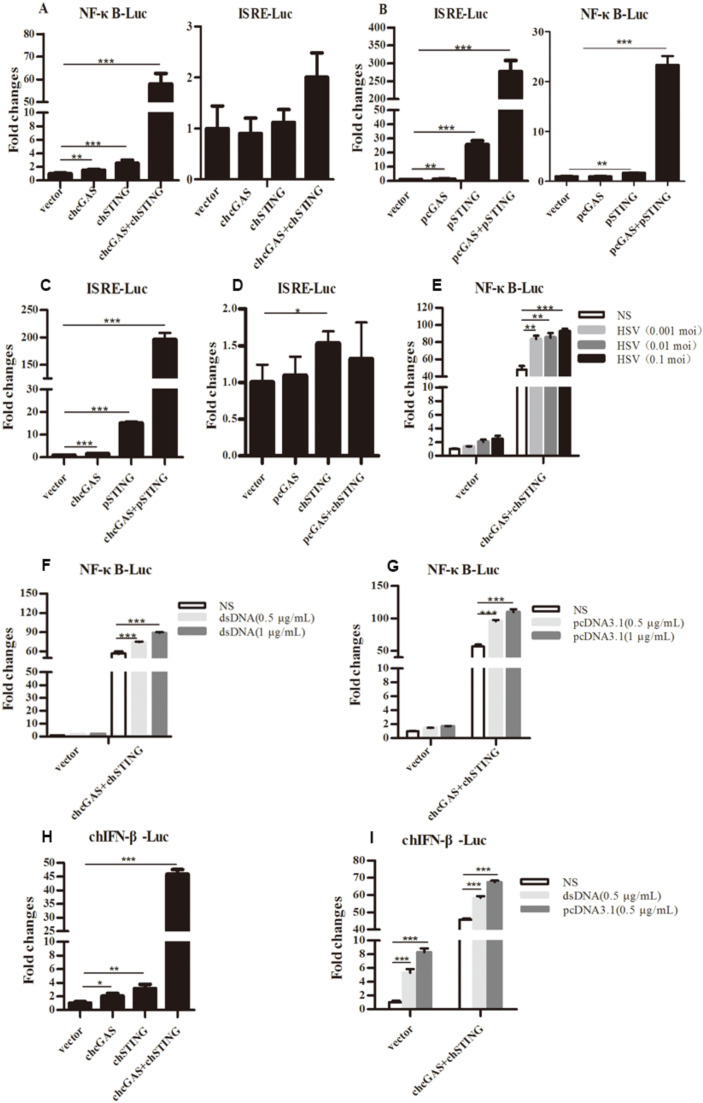
Promoter activation by chcGAS and chSTING. (**A–D**) 293T cells grown in 96-well plates (3 × 10^4^ cells/well) were transfected with chcGAS/pcGAS (20 ng), chSTING/pSTING (10 ng) plus ISRE-Fluc/ELAM-Fluc (10 ng) and Rluc (0.2 ng), which were normalized to 50 ng/well using the lipofectamine 2000. Twenty-four hours post transfection, the luciferase activities were measured with Double-Luciferase Reporter Assay. (**E–G**) 293T cells were transfected as in (**A–D**) and 24 h later were infected with HSV-1 or stimulated with dsDNA and pcDNA3.1 by transfection with the indicated concentrations. Eight to Twelve hours post stimulation, the luciferase activities were measured. (**H**,**I**) HD11 cells in 96-well plates were transfected as above with chIFNβ Fluc reporter using TransIT-LT1 Transfection Reagent, and 24 h later stimulated with dsDNA and pcDNA3.1 by transfection. Eight to Twelve hours post stimulation, luciferase activities were measured. The signs “*”, “**”, “***” denote *p* < 0.05, *p* < 0.01 and *p* < 0.001, respectively.

The signaling functions of chcGAS and chSTING were studied first in transfected 293T cells by promoter reporter gene assays. ChSTING on its own was able to weakly activate ELAM (NF-κB) promoter, whereas co-expression of chcGAS and chSTING strongly induced NF-κB activation (Figure 2A). Intriguingly, none of chcGAS and/or chSTING expression activated ISRE promoter reporter genes (Figure 2A). These phenotypes were in sharp contrast with mammalian cGAS and STING, where porcine cGAS and STING co-expression strongly activated ISRE promoter, as well as NF-κB promoter (Figure 2B). To address which protein plays a role in the ISRE promoter activation, we switched the cGAS and STING between chicken and porcine. The results showed that co-expression of chcGAS and pSTING was enough to strongly induce ISRE promoter activation, whereas co-expression of pcGAS and chSTING failed to activate ISRE promoter (Figure 2C,D), suggesting the critical role of the adaptor STING in activation of ISRE promoter. Upon infection by HSV-1 and transfections of dsDNA and pcDNA3.1, the chcGAS and chSTING induced NF-κB activities were significantly upregulated (*p* < 0.01, Figure 2E–G). The signaling function of chcGAS and chSTING was also assessed in chicken HD11 cells by chicken IFNβ promoter assay, and the results showed that chcGAS and chSTING co-expression strongly activated chIFNβ promoter and that the activity could be further heightened by stimulations with dsDNA and pcDNA3.1 (Figure 2H–I).

**Figure 3:**
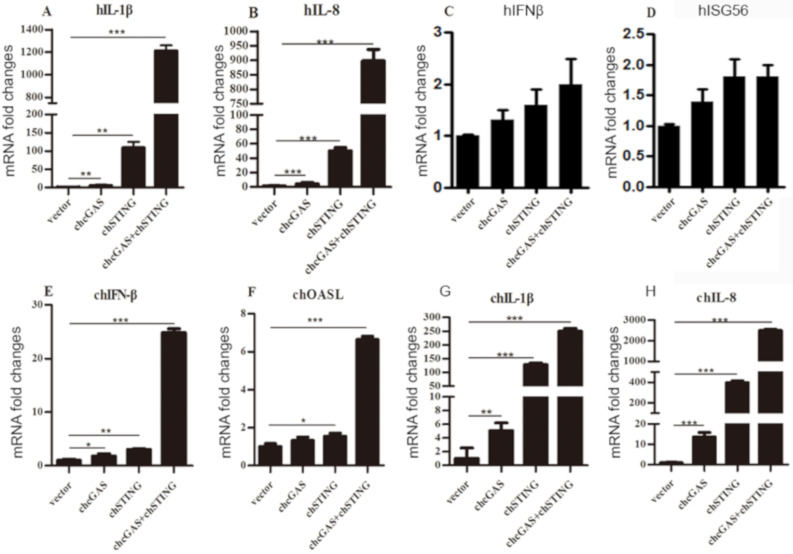
Cellular gene transcriptions activated by chcGAS and chSTING. (**A**–**D**) 293T cells grown in 12-well plates (4 × 10^5^ cells/well) were transfected with chcGAS (0.8 μg), chSTING (0.4 μg) or combination using lipofectiamine 2000. Twenty-four hours post transfection, the cells were analyzed by RT-qPCR for downstream gene expressions as indicated. (**E**–**H**) HD11 cells in 12-well plates (4 × 10^5^ cells/well) were transfected as in (**A**–**D**) using TransIT-LT1 Transfection Reagent, and 24 h later, cells were analyzed by RT-qPCR for downstream IFNβ, OASL, IL-1β and IL-8 gene expressions. The signs “*”, “**”, “***” denote *p* < 0.05, *p* < 0.01 and *p* < 0.001, respectively.

The cellular downstream gene expressions of transfected 293T and HD11 cells were examined by RT-qPCR. Co-expression of chcGAS and chSTING in 293T cells prominently induced the expressions of IL-1β and IL-8 (Figure 3A,B) but not IFNβ and ISG56 (Figure 3C,D), whereas, in transfected HD11 cells, the IFNβ, OASL, IL-1β, and IL-8 were all induced by co-expression of chcGAS and chSTING (Figure 3E–H). These results were consistent with and well reflected by those from promoter assays in 293T and HD11 cells. The signaling function of chcGAS-STING was also confirmed in DF-1 cells, where the chSTING activity was too strong to be observed for the synergy with chcGAS (Figure S6A,B).

### 3.2. Analysis and Identification of chcGAS Signaling Essential Sites

We next sought to compare chcGAS with mammalian cGAS counterparts, and chcGAS amino acid sequence was aligned with those of human, mouse and porcine cGAS using the software Clustal X as shown in Figure S1. The functional sites of human cGAS were predicted by UniProt (https://www.uniprot.org) and among the potential functional sites, those conserved across the 4 species were picked and marked in Figure S1. These functional conserved sites of chcGAS are S129, E141, D143, D239, E303, N309, H310, C317, C324, K334 and S353-K357, whose functions in human cGAS are involved in ATP, GTP, magnesium and zinc binding (Table 2).

**Figure 4:**
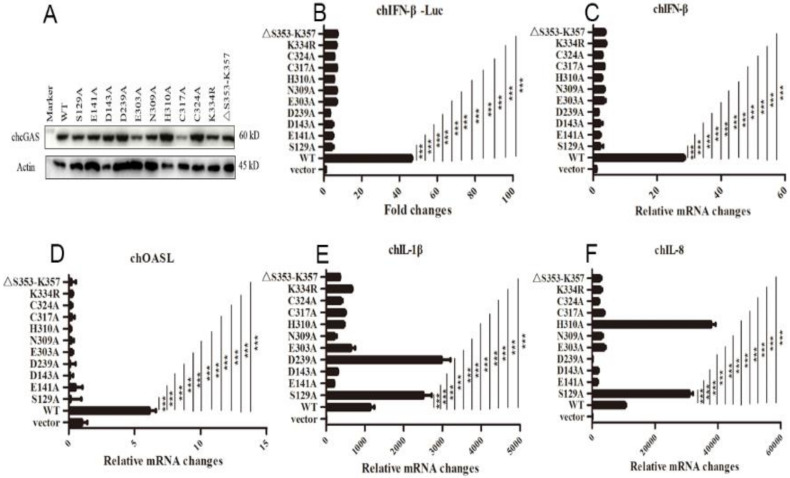
The expressions and activities of chGAS mutant proteins. (**A**) 293T in 24-well plates (2 × 10^5^ cells/well) were transfected with chGAS mutants (0.5 μg each), and 24 h later the cell lysates were analyzed by Western-blotting with anti-FLAG mAb. (**B**) Different chcGAS mutants (20 ng each) were co-transfected with chSTING (10 ng) plus chIFNβ Fluc reporters into HD11 cells in 96-well plate (3 × 10^4^ cells/well), and 24 h later, the cells were subjected to luciferase measurement. (**C**–**F**) HD11 cells in 12-well plate were co-transfected with chcGAS mutants (0.8 μg) and chSTING (0.4 μg), and 24 h later the cells were analyzed by RT-qPCR for cellular IFNβ, OASL, IL-1β and IL-8 gene transcriptions. The signs “***” denote *p* < 0.001.

The chcGAS mutants of these conserved sites were prepared and their protein expressions were confirmed by Western-blotting (Figure 4A). The signaling activities of these chcGAS mutants were examined by chIFNβ promoter assay in HD11 co-transfected with chSTING. The results showed that all these chGAS mutants lost the IFNβ promoter activity (Figure 4B). Consistently, the HD11 cellular downstream IFNβ and OASL productions also disappeared (Figure 4C,D). However, despite that most chcGAS mutants were unable to induce IL-1β and IL-8 productions, the IL-1β production by mutants S129A and D239A, and the IL-8 production by mutants S129A and H310A were actually higher than those induced by wild type chcGAS (Figure 4E,F), indicating the complexity of cGAS signaling.

### 3.3. chcGAS and chSTING Knockout (KO) and Overexpressing (OE) Stable HD11 Cells

**Figure 5:**
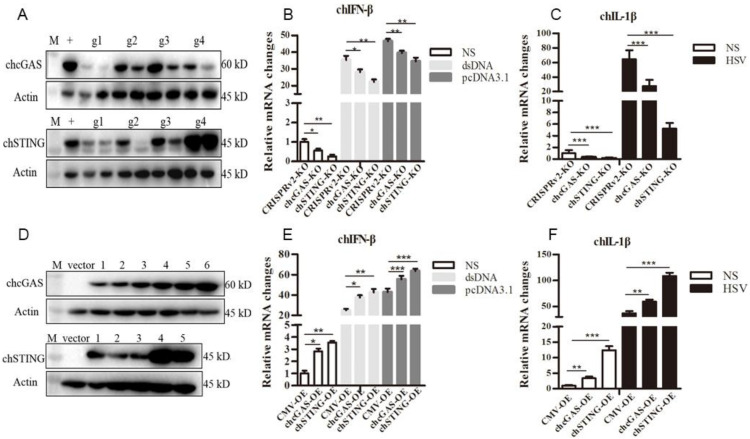
Establishment of chcGAS and chSTING KO and OE stable HD11 cells. (**A**) The efficacies of CRISPR gRNAs to suppress ectopic chcGAS and chSTING expressions. Four lentiviral gRNA plasmids of chcGAS (g1–g4, 0.25, 0.5 μg each) and chSTING (g1–g4, 0.25, 0.5 μg each) were co-transfected with chGAS-FLAG (0.5 μg) and chSTING-FLAG (0.5 μg), respectively, into 293T cells in 24-well plates. Twenty four hours later, cells were analyzed by Western-blotting with FLAG mAb for chcGAS and chSTING expressions. + denotes plentiCRISPRv2 vector plus chGAS or chSTING; M, protein marker. (**B**,**C**) The chcGAS KO, chSTING KO and pCRISPRv2 control HD11 cells in 12-well plates were stimulated with dsDNA (0.5 μg/mL), pcDNA3.1 (0.5 μg/mL) and Herpes Simplex Virus-1 (HSV-1) (0.01 MOI) for 812 h. The cells were analyzed by RT-qPCR for IFNβ and IL-1β transcriptions. (**D**) The different plasmids of plentiCMV-chcGAS (clones 1–6) and plentiCMV-chSTING (clones 1–5) were transfected into 293T cells, and, 24 h later cells, were analyzed by Western-blotting with anti-FLAG mAb for chGAS and chSTING expressions. The vector denotes plentiCMV control. (**E,F**) The chcGAS OE, chSTING OE, and pCMV control HD11 cells in 12-well plates were stimulated with dsDNA (0.5 μg/mL), pcDNA3.1 (0.5 μg/mL), and HSV-1 (0.01 multiplicity of infection, MOI) for 8-12 h. The cells were analyzed by RT-qPCR. The signs “*”, “**”, “***”denote *p* < 0.05, *p* < 0.01 and *p* < 0.001, respectively.

To study the roles of chcGAS-chSTING in the antiviral functions, the gene knockout (KO) and overexpressing (OE) stable HD11 cells were utilized. The lentiviral gRNA vectors were tested for knockout efficacy; based on the best efficacy, the gRNA1 was chosen for chcGAS and gRNA2 was for chSTING, respectively (Figure 5A). The resultant chGAS KO, chSTING KO, and CRISPR control stable HD11 cells were stimulated with dsDNA, pcDNA3.1, and HSV-1, HD11 cells responded to all the stimulations to produce cellular downstream gene transcriptions (Figure 5B,C and Figure S2). The downstream expressions of IFNβ, OASL, IL-1β and IL-8 after stimulations with dsDNA and pcDNA3.1, and expressions of IL-1β and IL-8 after stimulation with HSV-1 were all reduced in chcGAS KO and chSTING KO cells relative to control CRISPR HD11 cells (Figure 5B,C and Figure S2). The expressions of different lentiviral chcGAS and chSTING in transfected 293T cells were shown in Figure 5D. The clones with the highest expressions were used for virus package and infection of HD11 cells. Compared with the control stable CMV HD11 cells, the chGAS OE, chSITNG OE HD11 cells exhibited pronounced downstream gene transcriptions (Figure 5E,F and Figure S2). The chGAS OE, chSITNG OE and control CMV stable HD11 cells were stimulated with dsDNA, pcDNA3.1 and HSV-1, respectively. The expressions of IFNβ, OASL, IL-1β and IL-8 following dsDNA and pcDNA3.1 stimulations, and IL-1β and IL-8 expressions following HSV-1 stimulation were all increased in chcGAS OE and chSTING OE cells relative to control CMV HD11 cells (Figure 5E–F and Figure S2).

### 3.4. Anti-DNA Virus Functions by chcGAS and chSTING

**Figure 6:**
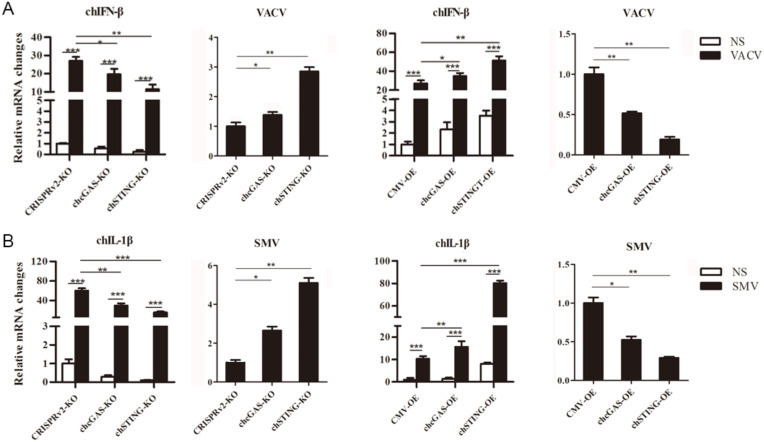
Vaccinia virus replications in chcGAS and chSTING stable HD11 cells. (**A**) HD11 KO and OE stable cells in 12-well plate (2 × 10^5^ cells/well) were infected with VACV (0.01 MOI) for 20 h and 20 h, respectively. The cells were analyzed by RT-qPCR for both cellular gene and viral gene transcriptions. (**B**) HD11 KO and OE stable cells in 12-well plate (2 × 10^5^ cells/well) were infected with SMV (0.01 MOI) for 20 h and 20 h, respectively. The cells were analyzed by RT-qPCR for both cellular gene and viral gene transcriptions. The signs “*”, “**”, “***” *p* < 0.05, *p* < 0.01 and *p* < 0.001, respectively.

To test the anti-DNA virus activity, chcGAS and chSTING stable HD11 cells were infected with Vaccinia Virus stains VACV and SMV, respectively. Upon VACV strain infection, the HD11 cells produced IFNβ, OASL, IL-1β, and IL-8 transcripts. In chcGAS KO and chSTING KO cells, the IFNβ, OASL, IL-1β, and IL-8 transcript productions were significantly (*p* < 0.05) reduced relative to control HD cells, whereas the viral C11R gene expressions were increased significantly (*p* < 0.05) relative to control HD11 cells (Figure 6A and Figure S3). On the other hand, in chGAS OE and chSTING OE cells, the IFNβ, OASL, IL-1β, and IL-8 transcript productions were significantly (*p* < 0.05) increased relative to control HD cells, whereas the viral C11R gene expressions were reduced significantly (*p* < 0.01) relative to control HD cells (Figure 6A and Figure S3). 

Upon SMV strain infection, the HD11 cells produced IL-1β and IL-8 but not IFN and ISG transcripts. In chcGAS KO and chSTING KO cells, the IL-1βand IL-8 transcript productions were reduced significantly (*p* < 0.01) relative to control HD11 cells, whereas the viral C11R gene expressions were increased significantly (*p* < 0.05) relative to control HD11 cells (Figure 6B and Figure S3). Conversely, in chGAS OE and chSTING OE cells, the IL-1βand IL-8 transcript productions were increased significantly (*p* < 0.01) relative to control HD cells, whereas the viral C11R gene expressions were reduced significantly (*p* < 0.05) relative to control HD cells (Figure 6B and Figure S3). The chcGAS and chSTING also exhibited similar anti-SMV activities in transfected DF-1 cells (Figure S6C). These results suggest that chcGAS and chSTING have very clear anti-DNA virus activity, as expected.

### 3.5. Anti-RNA Virus Functions by chcGAS and chSTING

**Figure 7:**
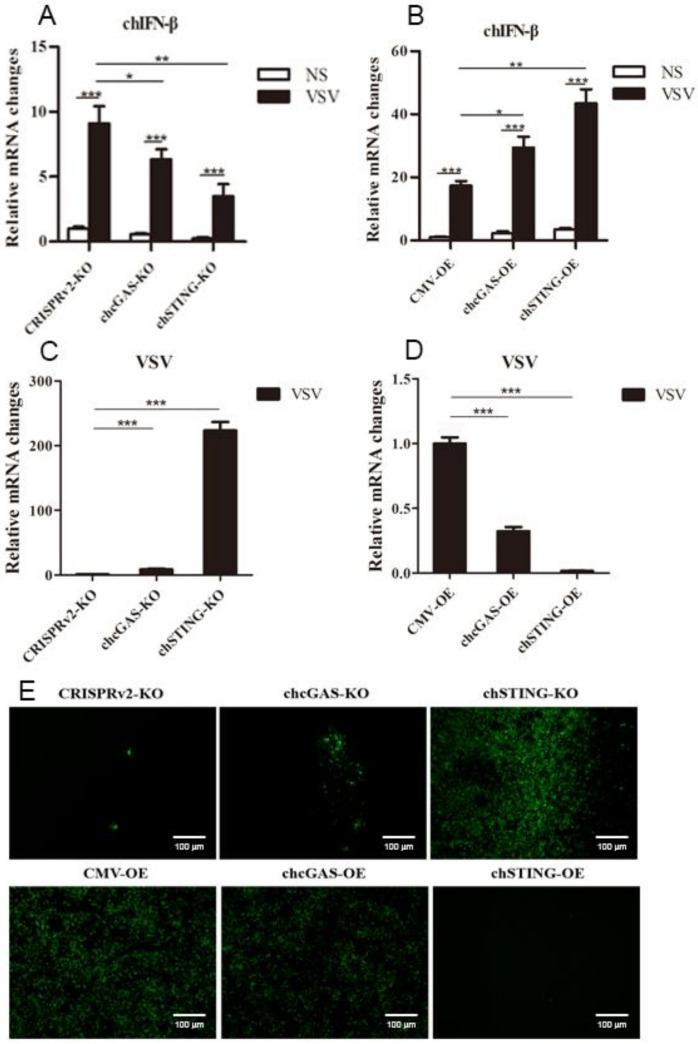
VSV-GFP replications in chGAS and chSTING stable HD11 cells. HD11 KO and OE stable cells in 12-well plate (2 × 10^5^ cells/well) were infected with VSV-GFP (0.01 MOI) for 14 h and 18 h, respectively. The cells were analyzed by RT-qPCR for both cellular gene transcriptions (**A,B**) and viral gene transcriptions (**C,D**). The viral replications in HD KO and OE cells were also monitored by GFP fluorescence microscopy (**E**). The signs “*”, “**”, “***” denote *p* < 0.05, *p* < 0.01 and *p* < 0.001, respectively.

**Figure 8:**
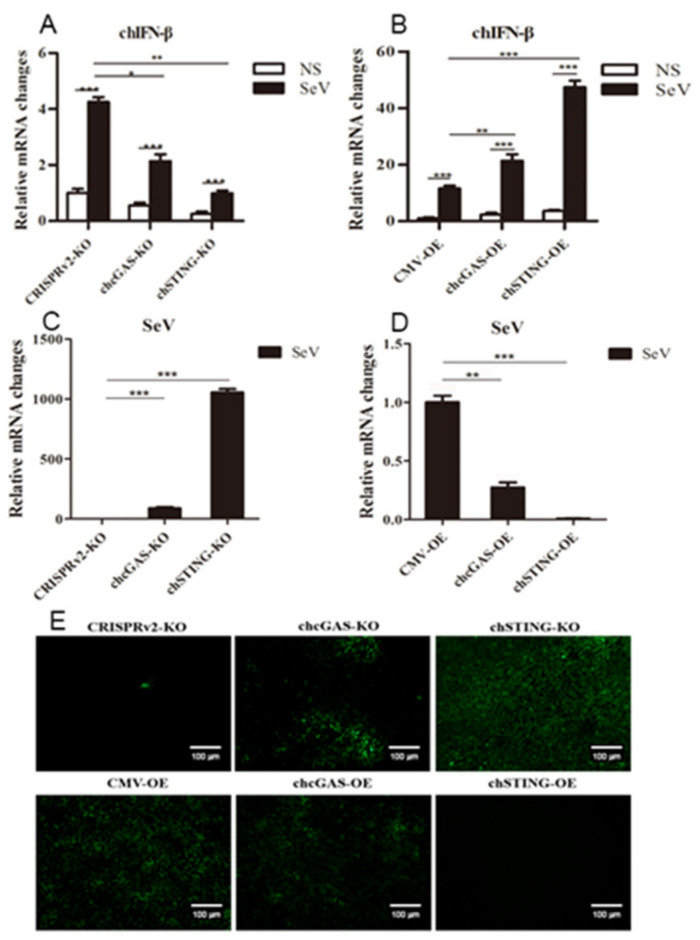
SeV-GFP replications in chGAS and chSTING stable HD11 cells. HD11 KO and OE stable cells in 12-well plate (2 × 10^5^ cells/well) were infected with SeV-GFP (0.01 MOI) for 14 h and 18 h, respectively. The cells were analyzed by RT-qPCR for both cellular gene transcriptions (**A,B**) and viral gene transcriptions (**C,D**). The viral replications in HD KO and OE cells were also monitored by GFP fluorescence microscopy (**E**). The signs “*”, “**”, “***” denote *p* < 0.05, *p* < 0.01 and *p* < 0.001, respectively.

**Figure 9:**
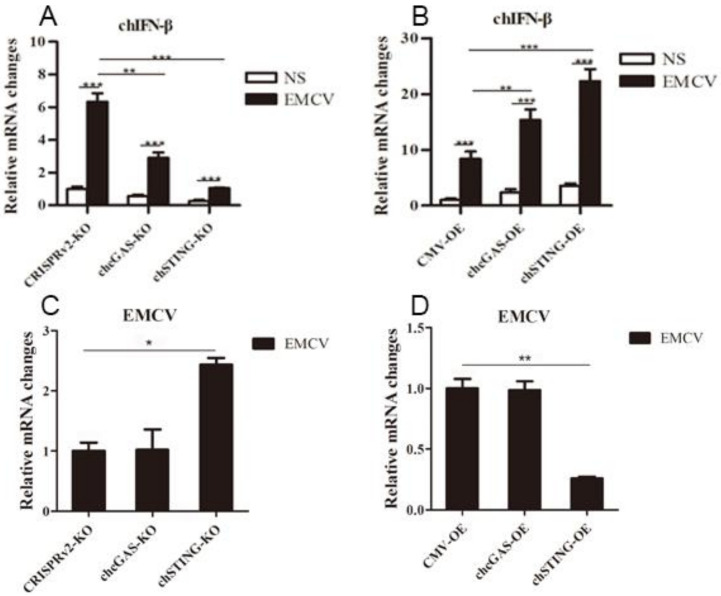
EMCV replications in chGAS and chSTING stable HD11 cells. HD11 KO and OE stable cells in 12-well plate (2 × 10^5^ cells/well) were infected with EMCV (0.01 MOI) for 14 h and 14 h, respectively. The cells were analyzed by RT-qPCR for both cellular gene transcriptions (**A,B**) and viral gene transcriptions (**C,D**). The signs “*”, “**”, “***” denote *p* < 0.05, *p* < 0.01 and *p* < 0.001, respectively.

The chcGAS and chSTING stable HD11 cells were infected with RNA viruses, including VSV-GFP, SeV-GFP, and EMCV. Upon the three RNA virus infections, the HD11 cells produced IFNβ, IL-1β, and IL-8 transcripts (Figure 7, Figure 8 and Figure 9 and Figure S4). In chcGAS KO and chSTING KO cells, the IFNβ, IL-1β, and IL-8 transcript productions were significantly (*p* < 0.05) reduced relative to control HD cells, whereas, in the chGAS OE and chSTING OE cells, the IFNβ, IL-1β, and IL-8 transcript productions were significantly (*p* < 0.05) enhanced compared with control HD cells (Figure 7A,B, Figure 8A,B, Figure 9A,B, and Figure S4). In terms of viral replications, VSV-GFP and SeV-GFP both exhibited significantly (*p* < 0.01) higher viral glycoprotein gene and GFP gene levels in chcGAS KO and chSTING KO cells, as well as significantly (*p* < 0.01) lower viral glycoprotein gene and GFP gene levels in chGAS OE and chSTING OE cells (Figure 7C–E and Figure 8C–E), suggesting the important roles of chcGAS-chSTING axis in anti-RNA virus replications. For EMCV replication, the viral polyprotein genes were significantly (*p* < 0.05) higher in chSTING KO cells and significantly lower (*p* < 0.01) in chSTING OE cells compared with the corresponding control HD11 cells (Figure 9C,D). However, their replications did not show any significant change in either chcGAS KO or chcGAS OE stable cells (Figure 9C,D).

### 3.6. Anti-Chicken NDV and Avian Retrovirus ALV Functions by chcGAS and chSTING

**Figure 10:**
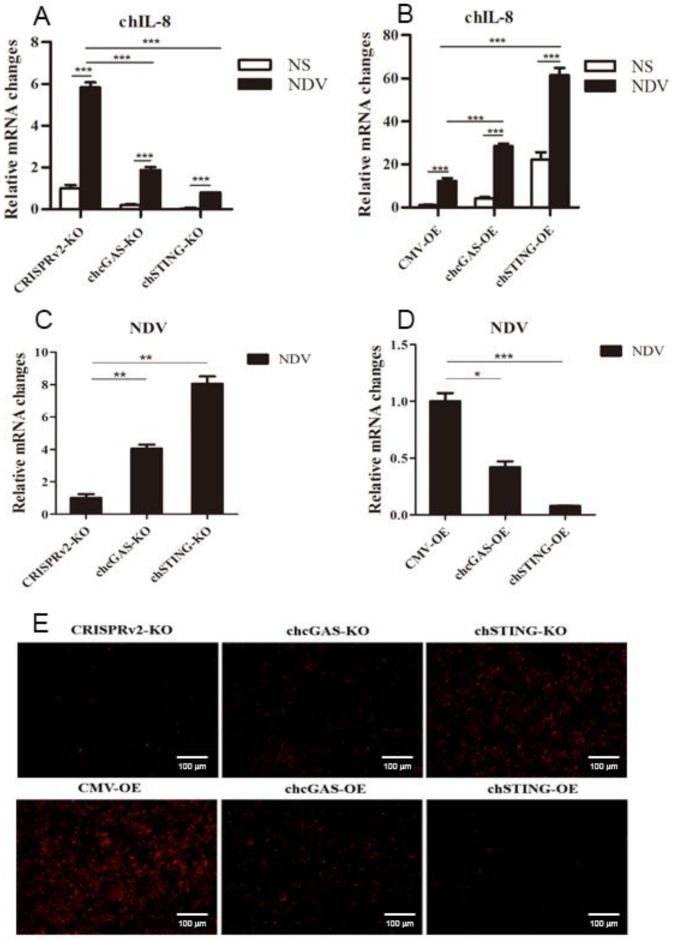
NDV-RFP replications in chGAS and chSTING stable HD11 cells. HD11 KO and OE stable cells in 12-well plate (2 × 10^5^ cells/well) were infected with NDV-RFP (0.01 MOI) for 8 h and 14 h, respectively. The cells were analyzed by RT-qPCR for both cellular gene transcriptions (**A,B**) and viral gene transcriptions (**C,D**). The viral replications in HD KO and OE cells were also monitored by RFP fluorescence microscopy (**E**). The signs “*”, “**”, “***” *p* < 0.05, *p* < 0.01 and *p* < 0.001, respectively.

**Figure 11:**
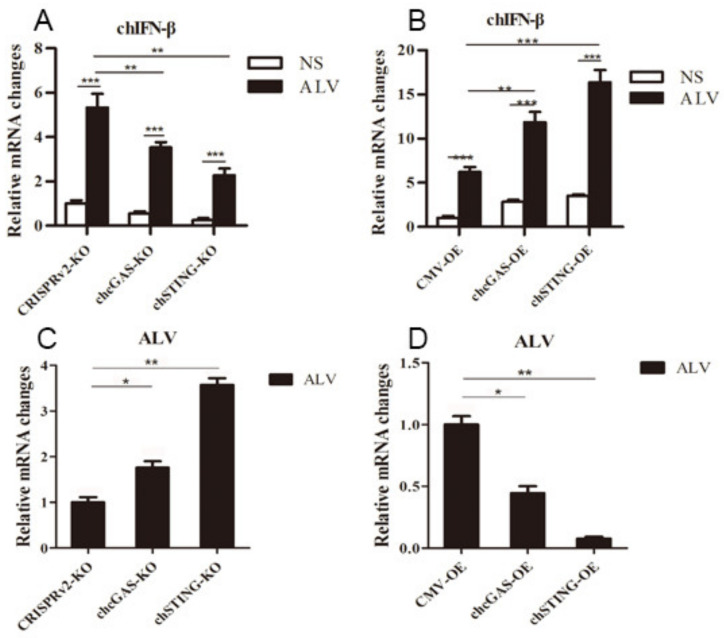
ALV-A replications in chGAS and chSTING stable HD11 cells. HD11 KO and OE stable cells in 12-well plate (2 × 10^5^ cells/well) were infected with ALV-A (0.01 MOI) for 14 h and 14 h, respectively. The cells were analyzed by RT-qPCR for both cellular gene transcriptions (**A,B**) and viral gene transcriptions (**C,D**). The signs “*”, “**”, “***” denote *p* < 0.05, *p* < 0.01 and *p* < 0.001, respectively.

To further demonstrate the antiviral functions of chcGAS and chSTING, we used the following relevant avian viruses, NDV and ALV-A, to infect the HD11 stable cells. Upon NDV-RFP infection, the virus-induced IL-8 transcript productions were significantly (*p* < 0.01) lower in chcGAS KO and chSTING KO HD11 cells and significantly (*p* < 0.01) higher in chcGAS OE and chSTING OE HD11 cells compared with the corresponding control HD11 cells (Figure 10A,B). Accordingly, the NDV showed significantly (*p* < 0.01) higher viral F gene and RFP expressions in both chcGAS KO and chSTING KO cells but significantly (*p* < 0.05) lower viral F gene and RFP expressions in both chcGAS OE and chSTING KO cells (Figure 10C–E). Upon ALV-A infection, the virus-induced IFNβ, IL-1β, and IL-8 transcript productions were significantly (*p* < 0.01) lower in chcGAS KO and chSTING KO cells but significantly (*p* < 0.01) higher in chGAS OE and chSTING OE cells compared with their corresponding control HD11 cells (Figure 11A,B, Figure S5). Consistently, ALV-A showed significantly (*p* < 0.05) higher viral Env gene expressions in both chcGAS KO and chSTING KO cells but significantly (*p* < 0.05) lower Env gene expressions in both chcGAS OE and chSTING OE cells compared with the corresponding control HD11 cells (Figure 11C,D).

## 4. Discussion

The species-specific differences in PRR sensing and signaling is of critical importance for immune biology, as well as for a suitable use of PRR agonists which possess immune modulatory activities and can thus be employed as vaccine adjuvants [27]. Despite to the low sequence identities with their mammalian counterparts (Table 1), the chcGAS-chSTING pathway appears to play a conserved and important role in sensing DNA and triggering the downstream IFN signaling. First, the co-transfection of chcGAS and chSTING in HD11 cells led to activation of chIFNβ promoter and chIFNβ, OASL, IL-1β, and IL-8 transcriptions (Figure 2H and Figure 3E–H). Second, chcGAS and chSTING are responsible for the cellular responses to dsDNA, pcDNA3.1, and HSV-1 stimulations (Figure 2I, Figure 5B–F, Figure S2). Third, the signaling competent NTase domains of chcGAS (AAs 80–428) and human cGAS (AAs 161–512) exhibited 69.2% similarity, a greater level of similarity than for the full length cGAS [36]. Similar to human cGAS, the chcGAS NTase domain adopts the typical NTase fold with a bilobed architecture comprising a N-terminal α/β catalytic core and a C-terminal helix bundle [37]. Corresponding to the conserved catalytic residues E225, D227, and D319 of human cGAS [27], the chicken E141, D143, and D239 are critical for chcGAS signaling to downstream type I IFN in transfected HD11 cells (Figure 4B–D). Furthermore, we showed that E141 is necessary for chcGAS to exert anti-VACV and VSV functions (Figure S6D,E). Regarding STING, the amino acids critical for the recognition of CDNs and the C-terminal pLxIS motif are remained conserved in both species (Figure S7), indicative of similar mechanisms of STING activation [38].

Nevertheless, species specificity of the cGAS-STING pathway exists, and differences are observed between birds and mammals. For example, chickens lack classical IRF3 but instead have homologous IRF7 compared with mammals [39]. A recent study discovered that chicken IRF7 constitutes IRF3 to mediate the relevant signaling function [30]; thus, like in mammals, the chicken cGAS-STING-TBK1-IRF7 signaling pathway plays a critical role in circumventing virus infection. In addition to IRF3, IRF9 seems also absent in chickens, but the substitution to mediate IFN signaling is still unknown [39]. In our study, we also found some differences in cGAS and STING between chickens and mammals. First, co-expression of chcGAS and chSTING in 293T cells did not activate ISRE promoter, instead inducing NF-κB activation, in sharp contrast with porcine cGAS and STING (Figure 2A,B). Further analysis of substitution of cGAS and STING between chicken and porcine species showed that STING plays a critical role in activating downstream ISRE signaling in 293T cells (Figure 2C,D). In line with this, and in contrast to human MAVS, chicken MAVS failed to activate human IFN-β promoter in 293T, despite its activity in avian cells [40]. The amino acids of phosphorylation and ubiquitination, and the critical pLxIS motif in human MAVS, are all missing in chicken MAVS, indicating distinct regulation of chMAVS [38,41]. However, the critical amino acids and the C-terminal pLxIS motif of STING are remained in both species (Figure S7). Therefore, the regions or amino acids determining the observed discrepancy would need to be identified and warrant further investigation.

Recent studies suggested chcGAS signaling plays a role in anti-Marek’s disease virus (MDV, member of the *Herpesviridae* family) and fowl adenovirus [31,33,34,42], both avian DNA viruses. However, the antiviral functions of chcGAS-chSTING pathway has not been fully defined. Here, we showed that chcGAS-chSTING exhibited antiviral functions against both DNA viruses (two Vaccinia strains) (Figure 6) and RNA viruses (VSV and SeV) (Figure 7 and Figure 8). For EMCV, another RNA virus, the chcGAS did not show any antiviral function, but chSTING did (Figure 9). The ectopic chcGAS tends to be lower activity compared with chSTING in our experiments; thus, the anti-EMCV function of chcGAS needs to be further examined and discerned using agonist stimulation and/or chcGAS^−/−^ cell clones. Additionally, chSTING not only mediates chcGAS signaling but also participates in chMDA5 signaling that senses viral RNA [43], which is also one reason why chSTING showed much more prominent anti-RNA virus activity than chcGAS. We also extended the antiviral effects of chcGAS and chSTING to physiological relevant avian RNA virus (NDV, Figure 10) and retrovirus (ALV-A, Figure 11), and the results showed that chcGAS-chSTING had antiviral functions against these two avian viruses. All the results pointed that chcGAS-chSTING signaling axis mediates broad spectrum antiviral effects against DNA viruses, RNA viruses, and Retroviruses. In this regard, the human cGAS has also been shown to mediate broad spectrum antiviral effects when assayed against 14 viruses representing 7 families and 11 genera [44].

In general, our work shows that the chicken cGAS-STING pathway has conserved signaling functions, but with species-specific distinctions, and mediates broad spectrum antiviral activities. The results indicate that the chicken cGAS-STING agonists, such as polydA:dT for cGAS and 2′-3′ cGAMP for STING, may be good immune adjuvants for chicken viral vaccines.

## 5. Conclusions

This study shows those points as following: chcGAS-chSTING is able to activate IFNβ in chicken cells but fails to activate IFN in mammalian cells. The conserved residues, including those in NTase domains of cGAS across four species, are essential for chcGAS signaling to IFN. chcGAS and chSTING harbor broad spectrum antiviral activities against DNA viruses, RNA viruses, and retrovirus.

## Figures and Tables

**Table 1:** Homology analysis of chcGAS and chSTING across species.

Genes	Chicken/Human	Chicken/Mouse	Chicken/Porcine
Identities of cGAS	48.5%	47.6%	49%
Identities of STING	42.6%	38.3%	43.3%

**Table 2:** The conserved sites and predicted functions in both chcGAS and hcGAS.

chcGAS Sites	Corresponding hcGAS Sites	Predicted Functions in hcGAS
S129	S213	ATP binding site
E141	E225	Magnesium binding site
D143	D227	Magnesium binding site
D239	D319	Magnesium, GTP binding sites
E303	E383	ATP binding site
N309	N389	Zinc binding site
H310	H390	Zinc binding site
C317	C397	Zinc binding site
C324	C404	Zinc binding site
K334	K414	ATP binding site
S353-K357	S435-K439	Magnesium, ATP binding region

## Supplementary Materials

The following are available online at https://www.mdpi.com/2076-393X/8/3/369/s1, Figure S1: Alignment of cGAS protein sequences across 4 species, Figure S2 (to Figure 5): The chcGAS KO, chSTING KO and pCRISPRv2 control HD11 cells in 12-well plates were stimulated with dsDNA (0.5 μg/mL), pcDNA3.1 (0.5 μg/mL) for 8–12 h (A–C) and HSV-1 (0.01 MOI) for 812 h (G). The chcGAS OE, chSTING OE and pCMV control HD11 cells in 12-well plates were stimulated with dsDNA (0.5 μg/mL), pcDNA3.1 (0.5 μg/mL) for 812 h (D–F) and HSV-1 (0.01 MOI) for 812 h (H). The cells were analyzed by RT-qPCR for cellular gene transcriptions, Figure S3 (to Figure 6): HD11 KO and OE stable cells in 12-well plate (2 × 10^5^ cells/well) were infected with VACV (0.01 MOI) for 20 h and 20 h, respectively (A–F). HD11 KO and OE stable cells in 12-well plate (2 × 10^5^ cells/well) were infected with SMV (0.01 MOI) for 20 h and 20 h, respectively (G–H). The cells were analyzed by RT-qPCR for cellular gene transcriptions, Figure S4 (to Figure 7, Figure 8 and Figure 9): HD11 KO and OE stable cells in 12-well plate (2 × 10^5^ cells/well) were infected with VSV-GFP (0.01 MOI) for 14 h and 18 h, respectively (A), SeV-GFP (0.01 MOI) for 14 h and 18 h, respectively (B), and EMCV (0.01 MOI) for 14 h and 14 h, respectively (C). The cells were analyzed by RT-qPCR for cellular gene transcriptions, Figure S5 (to Figure 11): HD11 KO (A,B) and OE (C,D) stable cells in 12-well plate (2 × 10^5^ cells/well) were infected with ALV-A (0.01 MOI) for 14 h and 14 h, respectively. The cells were analyzed by RT-qPCR for cellular gene transcriptions, Figure S6: The chcGAS anti-viral activity in DF-1 cells and the role of signaling essential site E141. (A) DF-1 cells in 96-well plate were transfected as in 293T cells, and the chIFNβ Luc was measured. (B) DF-1 cells in 24-well plate were transfected with chGAS and/or chSTING using TransIT-LT1 reagent; 24 h later the cells were infected with SMV (0.01 MOI) for 16 h. The downstream chIFNβ and SMV C11R gene transcriptions were measured by RT-qPCR. (D,E) HD11 cells in 12-well plate were transfected with chcGAS WT, E141A, or pcDNA vector (0.75 μg each) using TransIT-LT1 reagent; 24 h later the cells were infected with VACV and VSV for another 12 h. The viral gene expressions were measured by RT-qPCR, Figure S7: Alignment of STING protein sequences across 4 species. The GenBank accession numbers are: NP_938023.1 (human STING); NP_001136310.1 (porcine STING); NP_082537.1 (mouse STING); NP_001292081.1 (chicken STING). The alignment was generated by software Clustal X and drawn by BoxShade. Black shading indicates aa identity; gray shading indicates aa similarity (50%). The critical CDN binding amino acids and the C-terminal pLxIS motif of STING are marked with arrows and line, respectively. Table S1: The primers used for gene cloning and colony-PCR. Table S2: The PCR primers used for chcGAS mutations. Table S3: The CRISPR gRNA encoding DNA sequences. Table S4: Primers for RT-qPCR in this study.
