# Supplementary material for: Chicken DNA Sensing cGAS-STING Signal Pathway Mediates Broad Spectrum Antiviral Functions
Source: Vaccines (Basel). 2020 Jul 9;8(3):369. doi: 10.3390/vaccines8030369 (PMC7563795; doi:10.3390/vaccines8030369)
Supplement: Supplementary file 1 [file vaccines-08-00369-s001.pdf]

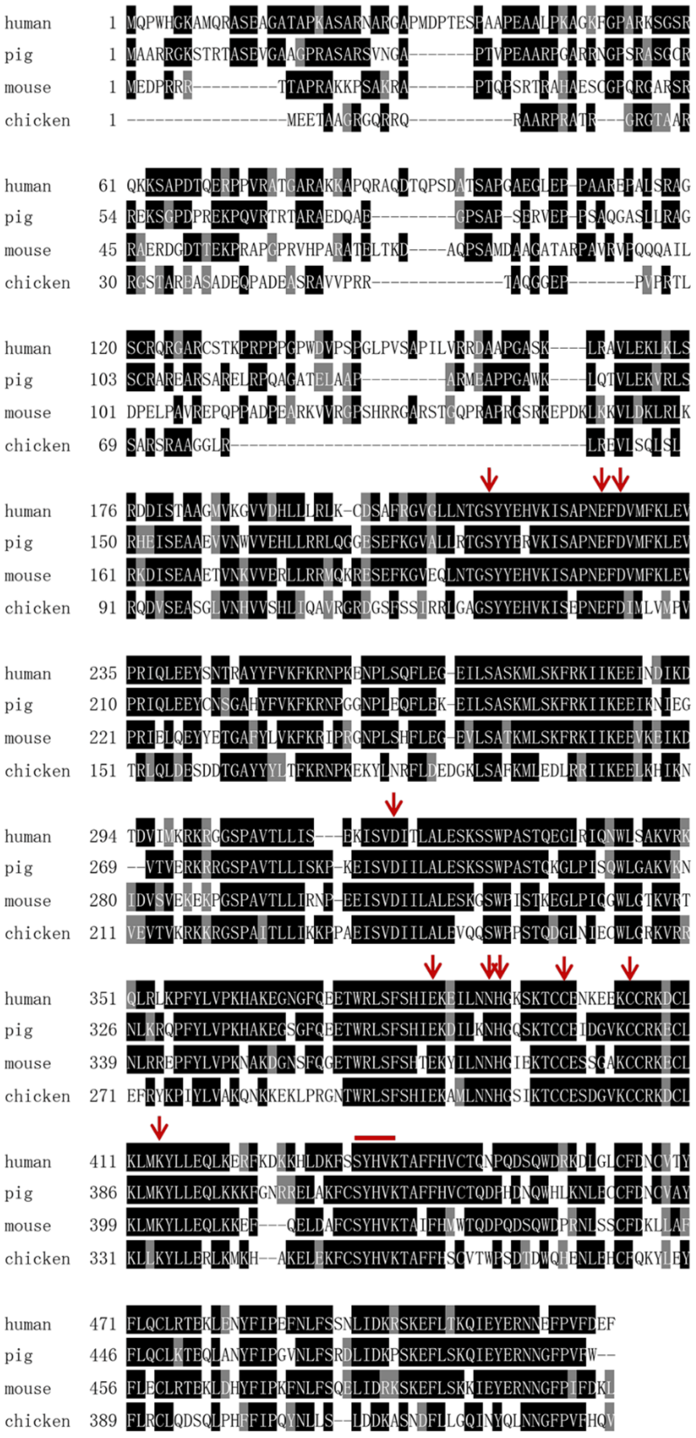

Figure S1. Alignment of cGAS protein sequences across 4 species. The GenBank accession numbers are: NP_612450 (human cGAS); XP_013840602.1(porcine cGAS); NP_775562.2 (mouse cGAS); XP_419881.4 (chicken cGAS). The alignment was generated by software Clustal X and drawned by BoxShade (https://embnet.vitalit.ch/software/BOX_form.html). Black shading indicates aa identity; gray shading indicates aa similarity (50%). The conserved functional sites for mutations were marked by arrows and line, respectively.

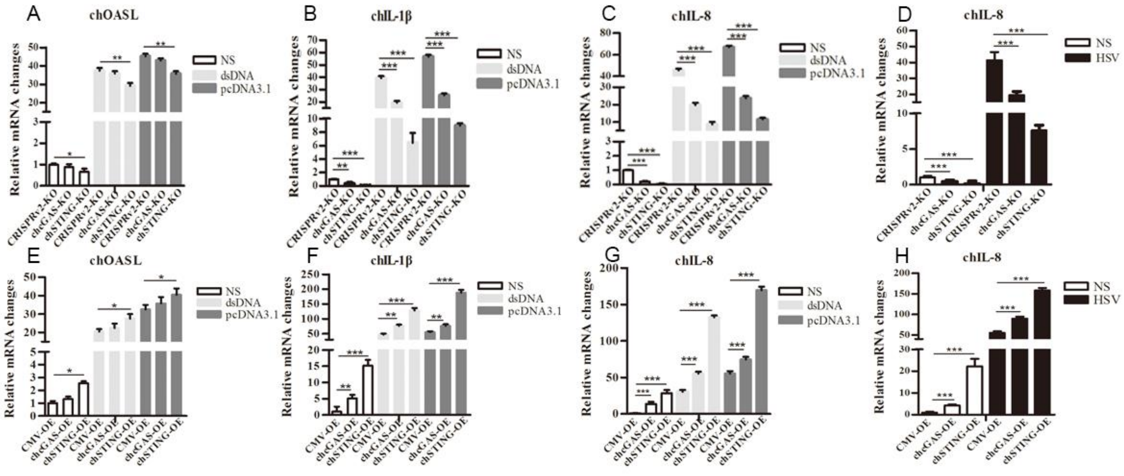

Figure S2 (to Figure 5). The chcGAS KO, chSTING KO and pCRISPRv2 control HD11 cells in 12-well plates were stimulated with dsDNA (0.5 μg/mL), pcDNA3.1 (0.5 μg/mL) for 8–12 h (**A–C**) and HSV-1 (0.01 MOI) for 8–12 h (**D**). The chcGAS OE, chSTING OE and pCMV control HD11 cells in 12-well plates were stimulated with dsDNA (0.5 μg/mL), pcDNA3.1 (0.5 μg/mL) for 8–12 h (**E–G**) and HSV-1 (0.01 MOI) for 8–12 h (**H**). The cells were analyzed by RT-qPCR for cellular gene transcriptions. "*", "**", "***" denote $p < 0.05$, $p < 0.01$ and $p < 0.001$, respectively.

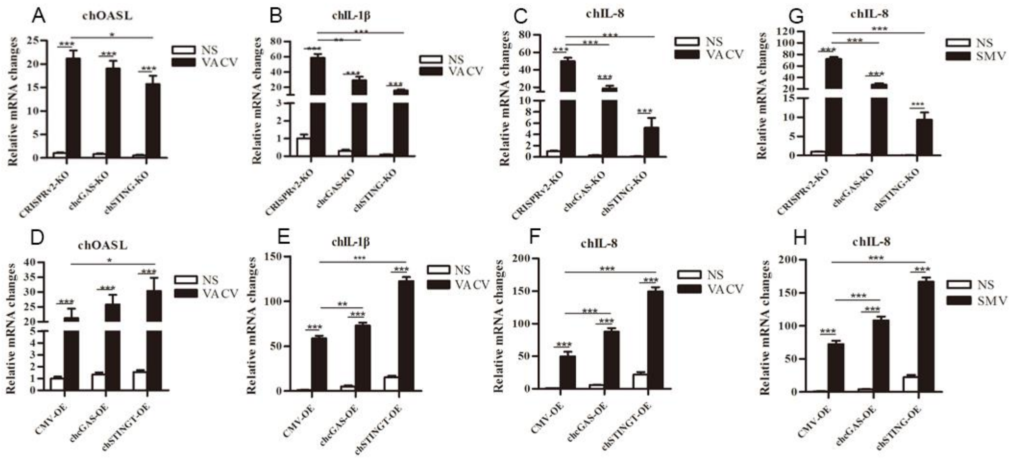

Figure S3 (to Figure 6). HD11 KO and OE stable cells in 12-well plate (2 × 10^5 cells/well) were infected with VACV (0.01 MOI) for 20 h and 20 h, respectively (**A–F**). HD11 KO and OE stable cells in 12-well plate (2 × 10^5 cells/well) were infected with SMV (0.01 MOI) for 20 h and 20 h, respectively (**G–H**). The cells were analyzed by RT-qPCR for cellular gene transcriptions. "*", "**", "***" denote $p < 0.05$, $p < 0.01$ and $p < 0.001$, respectively.

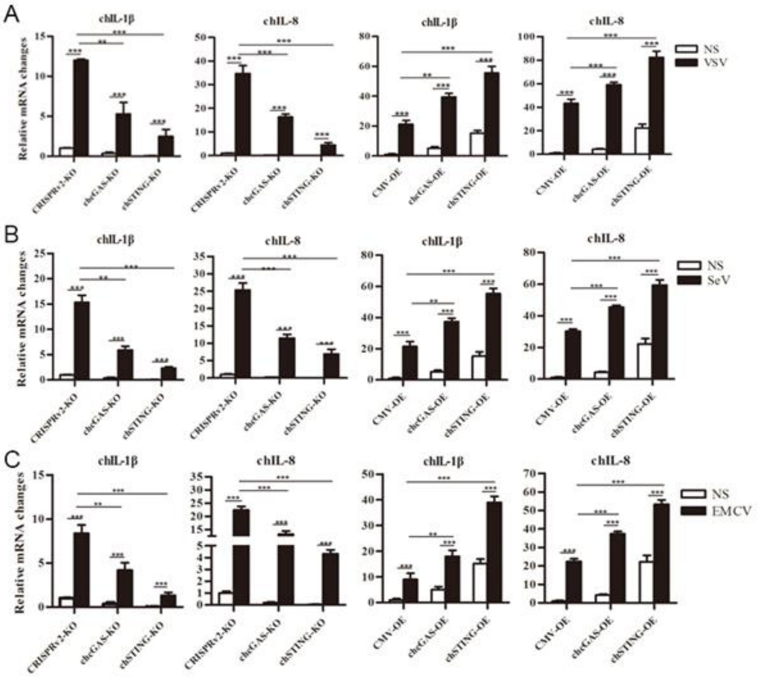

Figure S4. (to Figure 7–9). HD11 KO and OE stable cells in 12-well plate (2 × 10^5 cells/well) were infected with VSV-GFP (0.01 MOI) for 14 h and 18 h, respectively (**A**), SeV-GFP (0.01 MOI) for 14 h and 18 h, respectively (**B**), and EMCV (0.01 MOI) for 14 h and 14 h, respectively (**C**). The cells were analyzed by RT-qPCR for cellular gene transcriptions. "**", "***" denote $p < 0.01$ and $p < 0.001$, respectively.

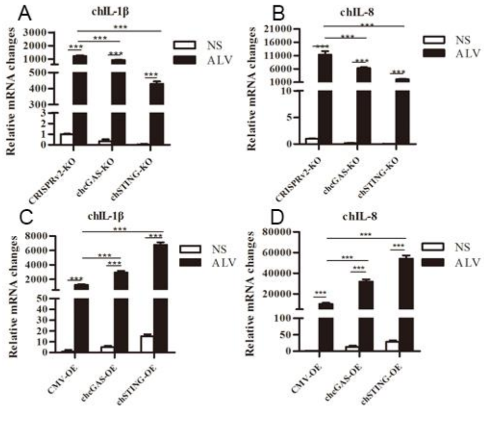

Figure S5 (to Figure 11). HD11 KO (**A–B**) and OE (**C–D**) stable cells in 12-well plate (2 × 10^5 cells/well) were infected with ALV-A (0.01 MOI) for 14 h and 14 h, respectively. The cells were analyzed by RT-qPCR for cellular gene transcriptions. "***" denotes $p < 0.001$.

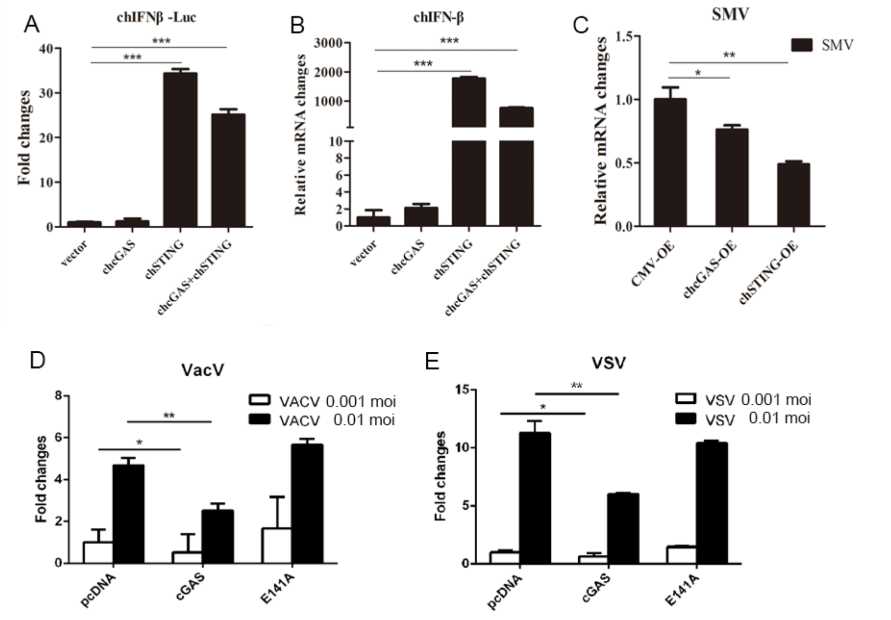

Figure S6. The chcGAS anti-viral activity in DF-1 cells and the role of signaling essential site E141. (**A**) DF-1 cells in 96-well plate were transfected as in 293T cells and the chIFNβ Luc was measured. (**B**) DF-1 cells in 24-well plate were transfected with chGAS and/or chSTING using TransIT-LT1 reagent, 24 h later the cells were analyzed for IFNβ by RT-qPCR. (**C**) The above transfected DF-1 cells were infected with SMV (0.01 MOI) for 16h. The SMV C11R gene transcription were measured by RT-qPCR. (**D–E**) HD11 cells in 12-well plate were transfected with chcGAS WT, E141A or pcDNA vector (0.75 μg each) using TransIT-LT1 reagent, 24 h later the cells were infected with VACV and VSV for another 12h. The viral gene expressions were measured by RT-qPCR. "*", "**", "***" denote $p < 0.05$, $p < 0.01$ and $p < 0.001$, respectively.

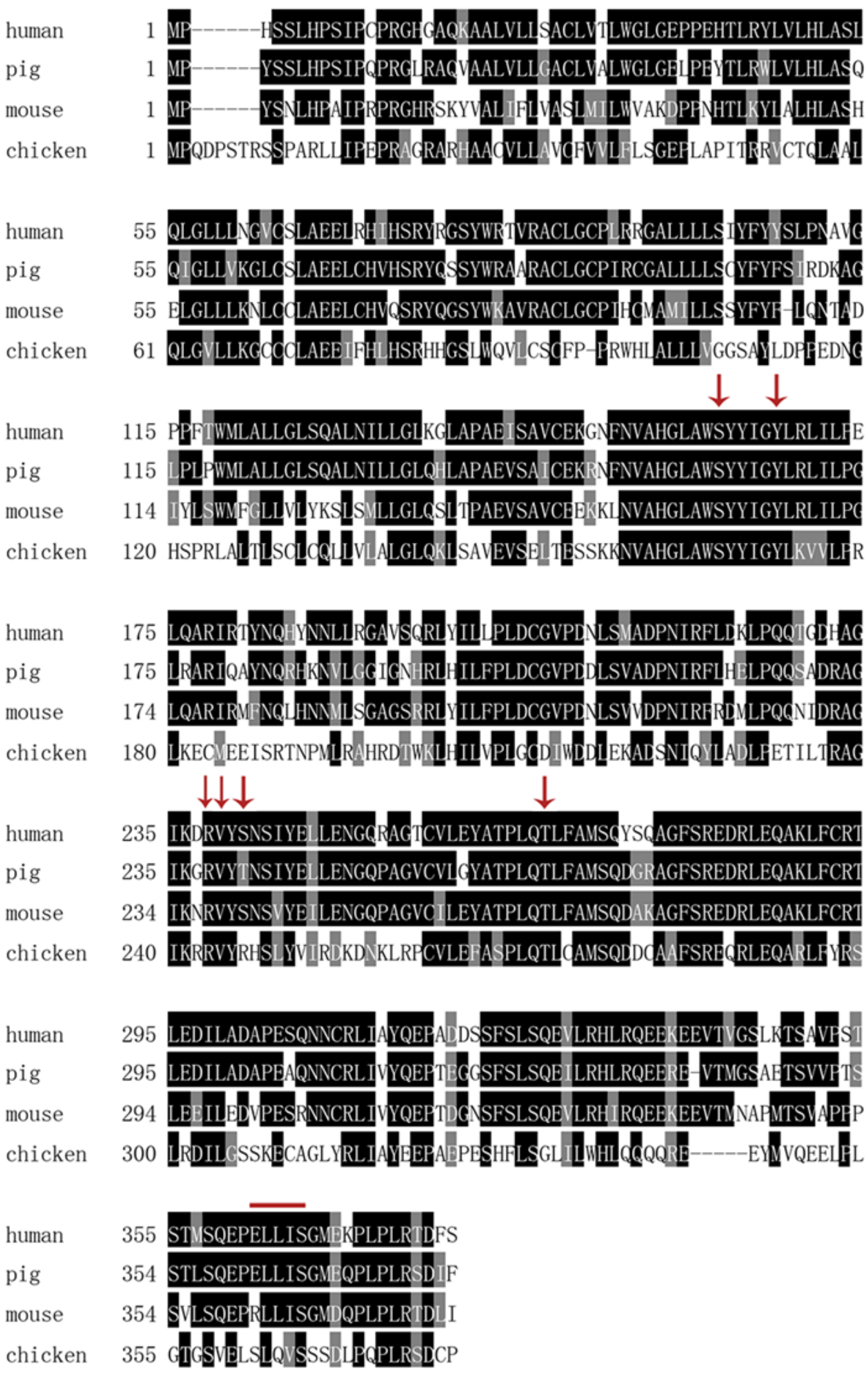

Figure S7. Alignment of STING protein sequences across 4 species. The GenBank accession numbers are: NP_938023.1 (human STING); NP_001136310.1 (porcine STING); NP_082537.1 (mouse STING); NP_001292081.1 (chicken STING). The alignment was generated by software Clustal X and drawned by BoxShade. Black shading indicates aa identity; gray shading indicates aa similarity (50%). The critical CDN binding amino acids and the C-terminal pLxIS motif of STING are marked with arrows and line, respectively.

Table S1. Primer sequences used for cloning and colony-PCR.

Genes Amplified	Primer Names	Primer Sequences (5′→3′)	Amplicon Sizes (bp)
chcGAS	chcGAS-F	GAGGCCCATGGAGGAGACCG	1332
	chcGAS-R	CCCGCCCCGGGCACCTGGTGAAAT ACTGGGAATCCATTGTTC	
	chcGAS-f	AAAGGAAAAAGCGTGGAAGCCC	253
	chcGAS-r	GAGAAAGAGAGTCGCCAGGTGT	
chSTING	chSTING-F	CCCGCGTCGACATGCCCCAGGACC CGTCAAC	1159
	chSTING-R	CCCGCGATATCGGGGCAGTCACTG CGCAG	
	chSTING-f	AAGAGATCAGCAGGACCAACCC	436
	chSTING-r	AAGAAGTGGCTCTCAGGCTCTG	

Note: The F/R denotes cloning primers with the underlined bases as restriction enzyme cut sites. The f/r denotes the colony-PCR primers for detection.

Table S2. Primer sequences used for mutation PCR of chcGAS.

chcGAS Mutation Sites	hcGAS Sites	Primer Names	Primer Sequences（5′–3′）
S129	S213	S_{129}A-F	GTGCTCGTAGTAGGCGCCGGCTCCCAGC
		S_{129}A-R	GCTGGGAGCCGGCGCCTACTACGAGCAC
E141	E225	E_{141}A-F	GCATTACAAGCATGATATCAAACGCATTTG GTTCAGATATCTTGACG
		E_{141}A-R	CGTCAAGATATCTGAACCAAATGCGTTTGA TATCATGCTTGTAATGC
D143	D227	D_{143}A-F	TAACAGGCATTACAAGCATGATAGCAAAC TCATTTGGTTCAGATATC
		D_{143}A-R	GATATCTGAACCAAATGAGTTTGCTATCAT GCTTGTAATGCCTGTTA
D239	D319	D_{239}A-F	TCCAAAGCCAAGATGATGGCCACTGATAT TTCTGCTG
		D_{239}A-R	CAGCAGAAATATCAGTGGCCATCATCTTG GCTTTGGA
E303	E383	E_{303}A-F	CTCTTTCTCGCATATCGCGAAGGCCATGCT GAACA
		E_{303}A-R	TGTTCAGCATGGCCTTCGCGATATGCGAGA AAGAG
N309	N389	N_{309}A-F	GTCTTTATGCTGCCGTGGGCGTTCAGCATG GCCTTCTC
		N_{309}A-R	GAGAAGGCCATGCTGAACGCCCACGGCAG CATAAAGAC
H310	H390	H_{310}A-F	GGCCATGCTGAACAACGCCGGCAGCATAA AGACG
		H_{310}A-R	CGTCTTTATGCTGCCGGCGTTGTTCAGCAT GGCC
C317	C397	C_{317}A-F	TTCACTCCATCGGATTCAGCGCACGTCTTT ATGCTGCC
		C_{317}A-R	GGCAGCATAAAGACGTGCGCTGAATCCGA TGGAGTGAA

C324	C404	$C_{324}A$-F	GAGACAATCTTTCCTGCAAGCCTTCACTCC ATCGGATTCA
		$C_{324}A$-R	TGAATCCGATGGAGTGAAGGCTTGCAGGA AAGATTGTCTC
K334	K414	$K_{334}R$-F	TTTTAAGTCGCTCTAGAAGATACCTCAGAA GTTTGAGACAATCTTTC
		$K_{334}R$-R	GAAAGATTGTCTCAAACTTCTGAGGTATCT TCTAGAGCGACTTAAAA
S353-K357	S435-K439	$\triangle S_{353}$-K_{357}-F	CATGAATGGAAAAAGGCAGTACAAAATTT TTCC AATTCTTTTGCATGTTTCATTTTAAG
		$\triangle S_{53}$-K_{357}-R	CTTAAAATGAAACATGCAAAAGAATTGGA AAAATTTTGTACTGCCTTTTTCCATTCATG

Table S3. CRISPR gRNA encoding DNA sequences (primers).

**Target Genes**	**gRNA Names**	**gRNA Primer Sequences (5′→3′)**
chcGAS	gchcGAS-1-F	CACCGTCGGCCGAGGCTTCCCGCG
	gchcGAS-1-R	AAACCGCGGGAAGCCTCGGCCGAC
	gchcGAS-2-F	CACCGTCCGCGCCGAGAGGGTTCGG
	gchcGAS-2-R	AAACCCGAACCCTCTCGGCGCGGAC
	gchcGAS-3-F	CACCGTCCCCCGAACCCTCTCGGCG
	gchcGAS-3-R	AAACCGCCGAGAGGGTTCGGGGGAC
	gchcGAS-4-F	CACCGAGGACGTGTCGGAGGCGTCG
	gchcGAS-4-R	AAACCGACGCCTCCGACACGTCCTC
chSTING	gchSTING-1-F	CACCGTGAGCCGCCGACAAGGAGCA
	gchSTING-1-R	AAACTGCTCCTTGTCGGCGGCTCAC
	gchSTING-2-F	CACCGGTAGGCTGAGCCGCCGACA
	gchSTING-2-R	AAACTGTCGGCGGCTCAGCCTACC
	gchSTING-3-F	CACCGAGCCTACCTGGACCCACCGG
	gchSTING-3-R	AAACCCGGTGGGTCCAGGTAGGCTC
	gchSTING-4-F	CACCGTGTGCCCATTGTCCTCCGGT
	gchSTING-4-R	AAACACCGGAGGACAATGGGCACAC

Table S4. The qPCR primers for cellular downstream genes and viral genes.

Genes Amplified	Primer Names	Primer Sequences (5′→3′)
hIFN-β	qhIFN-β-F	TGGGAGGATTCTGCATTACC
	qhIFN-β-R	CAGCATCTGCTGGTTGAAGA
hISG56	qhISG56-F	CGCTATAGAATGGAGTGTCCA
	qhISG56-R	TTTCCTCCACACTTCAGCA
hISG60	qhISG60-F	AGTCTAGTCACTTGGGGAAAC
	qhISG60-R	ATAAATCTGAGCATCTGAGAGTC
hIL-1β	qhIL-1β-F	TGAGGAAGATGCTGGTTCCCTG
	qhIL-1β-R	CCAGGAAGACGGGCATGTTTTC
hIL-8	qhIL-8-F	GTTTTTGAAGAGGGCTGAGAATTC
	qhIL-8-R	CATGAAGTGTTGAAGTAGATTTGCTTG
hIL-12	qhIL-12-F	CAGCAGTTGGTCATCTCTTGG
	qhIL-12-R	GGTCCAGGTGATACCATCTTCT
hRPL	qhRPL-F	CAACATTGGTTATGGAAGCAACA
	qhRPL-R	TGACGTTGTGGACCAGGAACT
chIFN-β	qchIFN-β-F	ATCTTCGTCACCAGGATGCCAA
	qchIFN-β-R	CGTGCCTTGGTTTACGAAGCAT
chOASL	qchOASL-F	CTGTCCTTCGGAGTCAGCATCA
	qchOASL-R	TCAGCAGCTCCAGTGCATACTT
chIL-1β	qchIL-1β-F	GTTTTTGAGCCCGTCACCTTCC
	qchIL-1β-R	CGGTAGAAGATGAAGCGGGTCA
chIL-8	qchIL-8-F	GGACGCTGGTAAAGATGGGGAA
	qchIL-8-R	CAGAATTGAGCTGAGCCTTGGC

chGAPDH	qchGAPDH-F	AGGGTGGTGCTAAGCGTGTTAT
	qchGAPDH-R	CAGCAGCCTTCACTACCCTCTT
VACV and SMV	qVWRC11-F	TCTGATGTTGTTGTTCGCTGCT
	qVWRC11-R	TCCATCTCCCTCTGGACCGCAT
VSV	qVSV-F	TGCAAGGAAAGCATTGAACAA
	qVSV-R	GAGGAGTCACCTGGACAATCACT
SeV	qSeV-F	GCAACATCCTGGGGCACAAGCT
	qSeV-R	TCGCCGACCACTACCAGCAGAA
EMCV	qEMCV-F	TCACCGTGAAGTCCGGCAGT
	qEMCV-R	TGTCAGACGCTGTGGCCTGA
NDV	qNDV-F	GGTCAATCATAGTCAAGTTGCTCC
	qNDV-R	AACCCCAAGAGCTACACTGCC
ALV	qALV-F	CTTTGGATTACATGGGCCGACC
	qALV-R	GAGACCTTCCGATAAGTGAGGG

Note: the hRPL and chGAPDH are the internal control genes in human and chicken cells, respectively.
